# Relationships Between Meaning in Life and Positive and Negative Spirituality in a Field Setting in Japan

**DOI:** 10.1007/s10943-023-01891-8

**Published:** 2023-08-19

**Authors:** Ryota Takano, Daiki Taoka

**Affiliations:** 1 Department of Social Psychology, The University of Tokyo, 7-3-1 Hongo, Bunkyo-Ku, Tokyo, 113-0033 Japan; 2 Japan Society for the Promotion of Science, Tokyo, Japan; 3 Graduate School of Education, Kyoto University, Kyoto, Japan

**Keywords:** Awe, Happiness, Meaning in life, Sadness, Spirituality

## Abstract

This study examined the relationships between positive/negative spirituality and meaning in life at two religious sites in Japan. Participants reported greater sadness and less meaning in life at Koyasan Okunoin, a temple with graves surrounded by nature, than at Nachi Kumano Taisha, a shrine with a waterfall. However, greater feelings of happiness protected meaning in life in the presence of sadness at Koyasan. Additionally, participants who felt a sense of awe reported greater meaning in life through self-liberation, regardless of the sites. The results provide new insights into meaning in life from the two sides of spirituality.

**Supplementary Information:**

The online version contains supplementary material available at 10.1007/s10943-023-01891-8.

## Introduction

Meaning in life is a critical component of well-being and mental/physical health (Boyle et al., 2010; Hill & Turiano, 2014; Kim et al., 2019; King et al., 2016; Steger, 2012; Yıldırım et al., 2021). Although its conceptualization varies across studies, there is a scholarly consensus that people experience a sense of meaning in life when they feel that they have (1) comprehension, (2) purpose, and (3) existential mattering in their lives beyond daily experiences (King & Hicks, 2021; King et al., 2006; Steger, 2012). Religiosity or spirituality is a vital source of meaning in life, as it provides clear guidelines on how to live under God or a higher power (Dar & Iqbal, 2019; Krause, 2003; Park, 2013). Previous research has demonstrated that spirituality has not only a positive side, but also a negative side, when God or a higher power punishes or abandons individuals (Exline et al., 2014; Jones et al., 2015; Pargament, 1997). This suggests that negative spiritual experiences might lead to the loss of a sense of meaning in life; however, little is known about how these two sides of spirituality are associated with meaning in life. Moreover, prior studies have involved procedures administered in laboratory or online settings with a focus on the propensity of spirituality, far from actual spiritual experiences. Thus, this study investigates the relationships between positive and negative spiritual experiences and meaning in life in a field setting with a particular focus on the Japanese context.

Spirituality is defined as a concern for or sensitivity toward immaterial things such as the spirit, which could include the practice of a particular religion (VandenBos & American Psychological Association, 2015). This definition is widely applicable to various cultures and religions, including those in Japan (Takano & Nomura, 2022a).^1^ Spiritual experiences cause individuals to perceive the unity of the world, thereby enhancing their comprehension and enabling them to recognize their life’s purposes and accept their existential mattering in life under God or a higher power, which increases their sense of meaning in life in all three above-mentioned components (Emmons, 2005; King & Hicks, 2021). A prior study that analyzed data from the Gallup World Poll of more than 450,000 individuals found that spirituality was associated with a greater sense of meaning in life (Diener et al., 2011; George & Park, 2017). In addition, experiences of spiritual or religious activities (e.g., meditation) increase one’s tendency to feel a sense of meaning in life (Pandya, 2019).

Contrastingly, spirituality’s negative aspect could cause one to lose meaning in life (Exline et al., 2014; Jones et al., 2015; Pargament, 1997). Spiritual experiences that are characterized by negative emotions, such as the experience of death, chaos, or isolation, can cause a sense of uncertainty in the world and destabilize one’s purpose and existential meaning. Previous studies have demonstrated that the sense of meaning in life is negatively associated with experiences of negative spirituality (Hicks & King, 2008; Krok, 2015). For example, negative spiritual experiences of patients with HIV (e.g., “I wonder if I have been abandoned by a higher-power”), or those facing chronic or life-threatening illnesses, are associated with lower levels of meaning in life (Szaflarski, 2013).

Positive affect or emotion (i.e., happiness) is an important predictor of meaning in life (Chu et al., 2020; King et al., 2006; Tov & Lee, 2016); critically, positive emotions play a role in protecting individuals from factors that interfere with meaning in life (Hicks & King, 2007, 2008, 2009). For example, feelings of positive emotions protect the sense of meaning in life even with low levels of religiosity and self-esteem, which are believed to be sources of meaning in life (Hicks & King, 2007, 2008, 2009). Meaning in life can be maintained by positive emotions even when experiencing negative spirituality.

Awe, an emotional response to conceptually and perceptually vast stimuli that transcend one’s current frames of reference (Keltner & Haidt, 2003), can emerge from both positive and negative spiritual experiences (Piff et al., 2015; Preston & Shin, 2017; Takano & Nomura, 2022b). Feelings of being small (non-significant), arising from a negative sense of awe, can be associated with less meaning in life (Rivera et al., 2020). However, simultaneously, self-liberation, a psychological process by which one can liberate and disengage from the self, is also believed to be a process involved in awe (Takano & Nomura, 2021, 2022b). Given that this self-liberation process is thought to be associated with the increase in accessibility to self-transcendence, the authentic self, and the true self (Frankl, 1992; Jiang & Sedikides, 2022; Van Cappellen & Rimé, 2013; Van Cappellen & Saroglou, 2012), feelings of awe during a spiritual experience are likely to be related to a higher sense of meaning in life through self-liberation.

### The Current Study

**Fig. 1:**
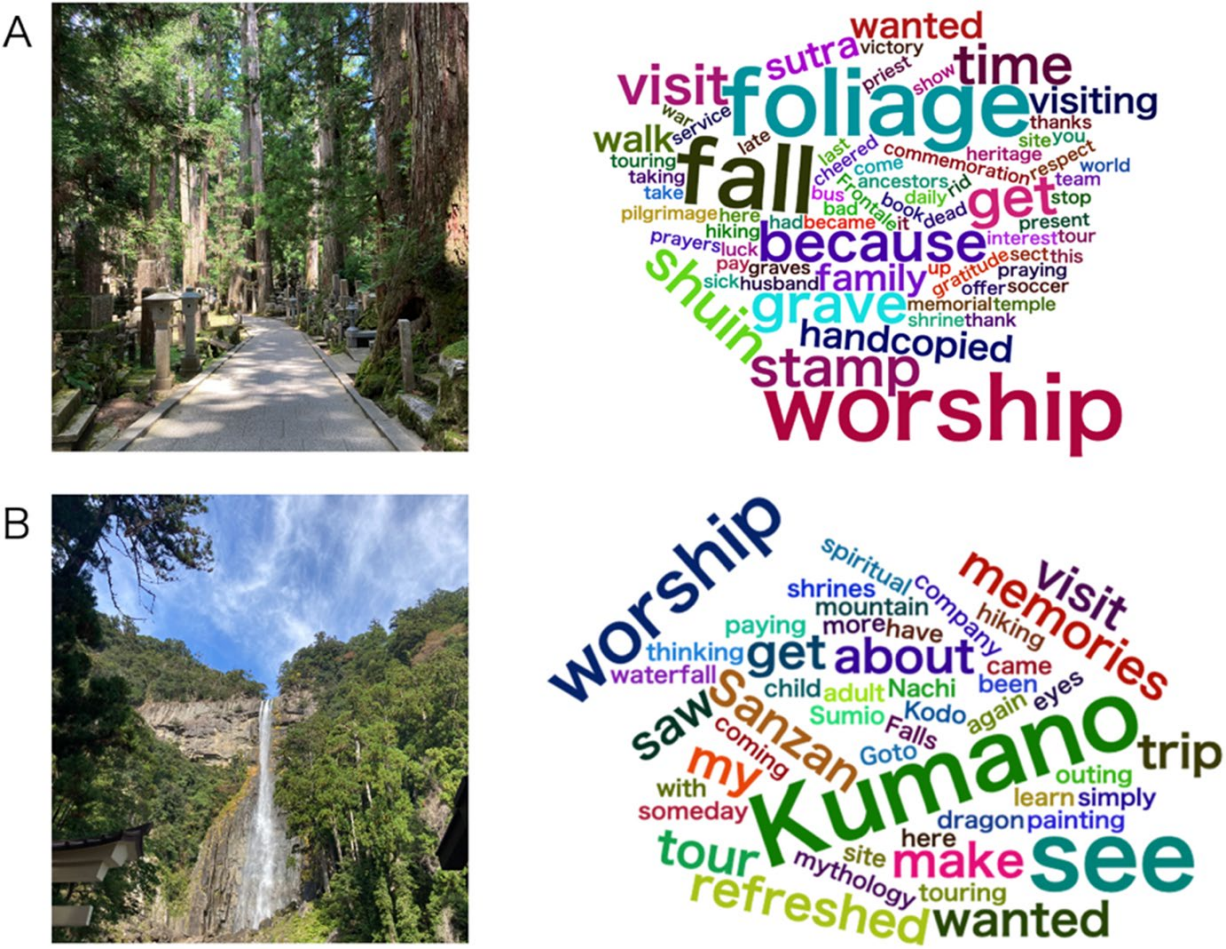
**a** Photographs and Frequencies of Words Used to Describe the Purpose of the Visit to Koyasan Okunoin Temple and **b** Kumano Nachi Taisha Shrine. *Note*. Bigger words indicate higher frequency. The word “sightseeing” was excluded to present the other words since it was the most frequent word in both conditions. *Shiun* is a seal stamp to indicate that a person has visited a shrine or temple. *Sanzan* is the collective name for three shrines: Kumano Nachi Taisha, Kumano Hongu Taisha, and Kumano Hayamizu Taisha

This study examined the relationships between positive and negative spiritual experiences and meaning in life at two famous religious sites in Japan. We conducted a field study among tourists at the Koyasan Okunoin temple and the Kumano Nachi Taisha shrine (Fig. 1a and b). Although these two religious sites have different religious and historical backgrounds, both are famous religious facilities in Japan; are registered as UNESCO World Natural Heritage sites; and have similar religious beliefs typically observed in Japan, which focus on being tolerant of other religions and open to non-religious people’s naïve religious and spiritual practices.

Koyasan is a sacred Buddhist site in Japan and was originally founded by Kūkai (also known as Kōbō Daishi). He established Shingon Buddhism, which focuses on the concept of “Sokushinjōbutsu (attainment of Buddhahood in this very body).” This concept indicates that everyone in the secular world can discover the sacredness within mundane reality that transcends specific beliefs and religions. Thus, the religious belief cultivated in Koyasan is characterized by a mixture of sacred and secular forms, embracing different beliefs and religions (Matsunaga, 2014; although it was historically restricted to women, similar to other sanctified mountains in Japan, such restrictions have been lifted). Consistent with this religious belief, the Koyasan Okunoin temple has approximately 200,000 graves of numerous people who had different ideologies and beliefs, including Kūkai, in nature (surrounded by a forest), where people visit their families’ and ancestors’ graves.

The Kumano Nachi Taisha shrine is part of the Kumano Sanzan, including the Kumano Hayatama Taisha and the Kumano Hongu Taisha shrines, registered as a UNESCO World Natural Heritage site as sacred places and pilgrimage routes in the Kii Mountain. The religious belief cultivated in this shrine is characterized by the syncretism of Shintoism and Buddhism, which is also associated with nature worship as it has the largest one-step waterfall in Japan. The waterfall has been regarded as a sacred object since the fourth century, when Emperor Jimmu, the first emperor of Japan, is said to have seen something sparkling in the mountains when his boat landed on the Kumano shore. Many tourists visit the shrine to get a spiritual force of the waterfall that heals or refreshes in addition to worshipping the gods.

In addition to these religious and historical backgrounds, the two sites are surrounded by magnificent nature, which evokes “naïve” religious and spiritual experiences for many spiritual but not religious Japanese people who are familiar with nature worship (Sugimoto, 2020). Hence, this study investigated “general” psychological processes of these experiences, including awe, by focusing on tourists rather than specific religious practitioners. Since the Koyasan Okunoin temple has numerous graves surrounded by nature, thereby involving spiritual components related to the death of relatives and ancestors, spiritual experiences at the Koyasan Okunoin temple are thought to involve negative components compared to experiences at the Kumano Nachi Taisha shrine. Thus, we tested the following three hypotheses:

#### H1

Participants at the Koyasan Okunoin temple would report higher levels of negative emotions and lower levels of meaning in life than those at the Kumano Nachi Taisha shrine.

#### H2

Participants who felt more positive emotion at the Koyasan Okunoin temple would maintain their meaning in life even when they felt negative emotion.

#### H3

Regardless of the type of religious site, participants who felt awe would exhibit higher levels of meaning in life through the feelings of self-liberation.

In addition to these three hypotheses, we explored different characteristics between the two sites by examining text-mining analyses and correlation analyses among variables.

## Method

### Participants

Participants included 98 Japanese visitors walking through the outside parking places at the gate of Koyasan Okunoin temple and Kumano Nachi Taisha shrine (66% women, mean age = 49.74 years, standard deviation [SD] = 17.81). Both participants at Koyasan Okunoin temple (i.e., the Koyasan condition) and Kumano Nachi Taisha shrine (i.e., the Nachi condition) comprised 49 tourists (Table 1 for demographic details). Our target sample size was approximately 100 as we could not predict how many tourists would participate in this study. After the completion of data collection, we conducted a post hoc power analysis to determine whether we had adequate power to detect between-groups differences among the ratings of sadness (Cohen’s *d* = 0.63) with the 98 participants included in our analyses, indicating that we had 87% power with *α* = 0.05 between the groups using a two-sample *t* test. The local institutional review board approved this study (CPE-439), and all participants provided written informed consent prior to their participation.

**Table 1:** Participants’ demographic characteristics

Demographic	Classification	Percentage or mean	
		Koyasan condition (*n* = 49)	Nachi condition (*n* = 49)
Age		49.37 years	50.12 years
Gender	Men	43%	24%
	Women	57%	76%
Religious affiliation	Shinto	4%	2%
	Buddhism	53%	29%
	Other	6%	0%
	No	35%	61%
	Not applicable	2%	8%

### Materials and Procedure

At each location, participants who seemed to come out of the shrine or temple were approached by research staff who asked if they could participate in the survey.

#### Measures

The following measures were used in this study.^2^ These measures of were adapted from validated and standardized questionnaires developed in previous studies (e.g., Steger et al., 2006; Takano & Nomura, 2022a). All items were rated on seven-point Likert scales (more details are available at https://osf.io/m95pn/).

**Sense of meaning in life.** To assess the sense of meaning in life, participants were asked to rate their agreement with a single statement, “I feel my life is meaningful,” adopted from the World Health Organization Quality of Life scale (World Health Organization Quality of Life Group, 1998). We used one item to minimize the time required to complete the survey.

**Emotion.** Participants reported the degree that they experienced nine emotional states such as happiness, sadness, and awe-related emotions (Takano & Nomura, 2022b).

**Self-liberation.** To assess the sense of self-liberation, participants were asked to rate their agreement with the statement, “I feel liberated from myself and the things engaging myself” (*M* = 4.22, SD = 1.54). We created this question based on the item in (Jiang et al., 2018) self-transcendence scale (“I can move beyond things that once seemed so important”) to capture the essence of the self-liberation process of awe.

**Feelings of spirituality.** To assess the feelings of spirituality, participants were asked to rate their agreement with the following statements, “I feel ‘something invisible’ (something larger than yourself, something grand, something transcendent),” “I feel connected with ‘something invisible’,” and “I feel that all life is interconnected” (modified from Piedmont, 1999; Takano & Nomura, 2022a; *α* = 0.61; *M* = 4.46, SD = 1.25). We used this questionnaire to confirm that places can induce a spiritual feeling.

**Purpose of the visit.** Participants were asked to provide a short descriptive answer to a question regarding the purpose of their visit to the place, “What is the purpose of your visit?”.

**Demographic questionnaire.** Participants were asked to state their age, gender, and religious affiliations (Table 1).

### Statistical Analyses

Data analyses were conducted in R (v 4.2.3) using Bayesian linear regression models (similar results were obtained using frequentist *t* tests and regression analyses; Supplemental Materials and Tables S1 and S6–S7). The largest amount missing for a single variable was 5.1%; thus, missing data were handled using multiple imputation method. First, to test *H1*, we determined whether the subjective ratings differed between the Koyasan and the Nachi conditions. Second, to test *H2*, the interaction effect between condition, sadness, and happiness on meaning in life were examined. Third, to test *H3*, the interaction effect between awe-related emotions (*ikei*, *ifu*) and self-liberation on meaning in life were examined, controlling for the main effect of condition. The values of all independent variables in the regression models with interactions were centered except the condition (0 = Nachi, 1 = Koyasan). When the interaction effect was observed, we calculated simple slopes using the emmeans package (Lenth et al., 2019). A coefficient was deemed significant when 95% posterior credible intervals (CIs) did not include 0, which was supplemented with 90% posterior CIs when the 95% CIs included 0. When 90% posterior CIs did not include 0, the results were reported as a trend (i.e., marginally significant, Hulsman et al., 2021; Makowski et al., 2019). Fourth, to explore the differences in the purpose of the visit to the two sites, text data mining methods were used for participants’ descriptions of the purpose of the visit to illustrate the frequency of the words for each place using the word segmentation with RMeCab package (Ishida, 2019) and visual description with the wordcloud2 package (Lang et al., 2018).^3^ We also conducted correlation analyses to exploratorily examine different relationships among variables between conditions (Tables S2 and S3). The data and the analysis script are available at https://osf.io/m95pn/.

## Results

First, the Bayesian logistic regression models revealed that there were (marginally) significant differences in distributions for gender (0 = male, 1 = female, 2 = other) and religious affiliation (0 = non-religious, 1 = religious) between conditions (gender [female]: *b* =  − 0.42, 90% CI [− 0.79, − 0.06], religious affiliation: *b* = 0.63, 95% CI [0.20, 1.06]). Therefore, we also investigated whether similar results of the following analyses were obtained when controlling for gender and religious affiliation.

Regarding the differences of subjective ratings between conditions, in line with *H1*, the Bayesian linear regression models showed that participants in the Koyasan condition reported a marginally lower sense of meaning in life than those in the Nachi condition (*M*_Koyasan_ = 5.24, *M*_Nachi_ = 5.71, *b* =  − 0.24, 90% CI [− 0.47, − 0.01], 95% CI [− 0.51, 0.03]). In addition, participants in the Koyasan condition reported greater feelings of sadness than those in the Nachi condition (*M*_Koyasan_ = 3.06, *M*_Nachi_ = 2.10, *b* = 0.48, 95% CI [0.18, 0.79]). Further, the mean ratings of the feelings of spirituality in both conditions were above midpoint (i.e., 4; Koyasan condition: *M*_Koyasan_ = 4.46, *b* = 0.44, 95% CI [0.05, 0.84], Nachi condition: *M*_Nachi_ = 4.46, *b* = 0.48, 95% CI [0.15, 0.82]), indicating that both places induced feelings of spirituality. There were no significant differences of other subjective ratings between the two conditions (Table S1).

**Fig. 2:**
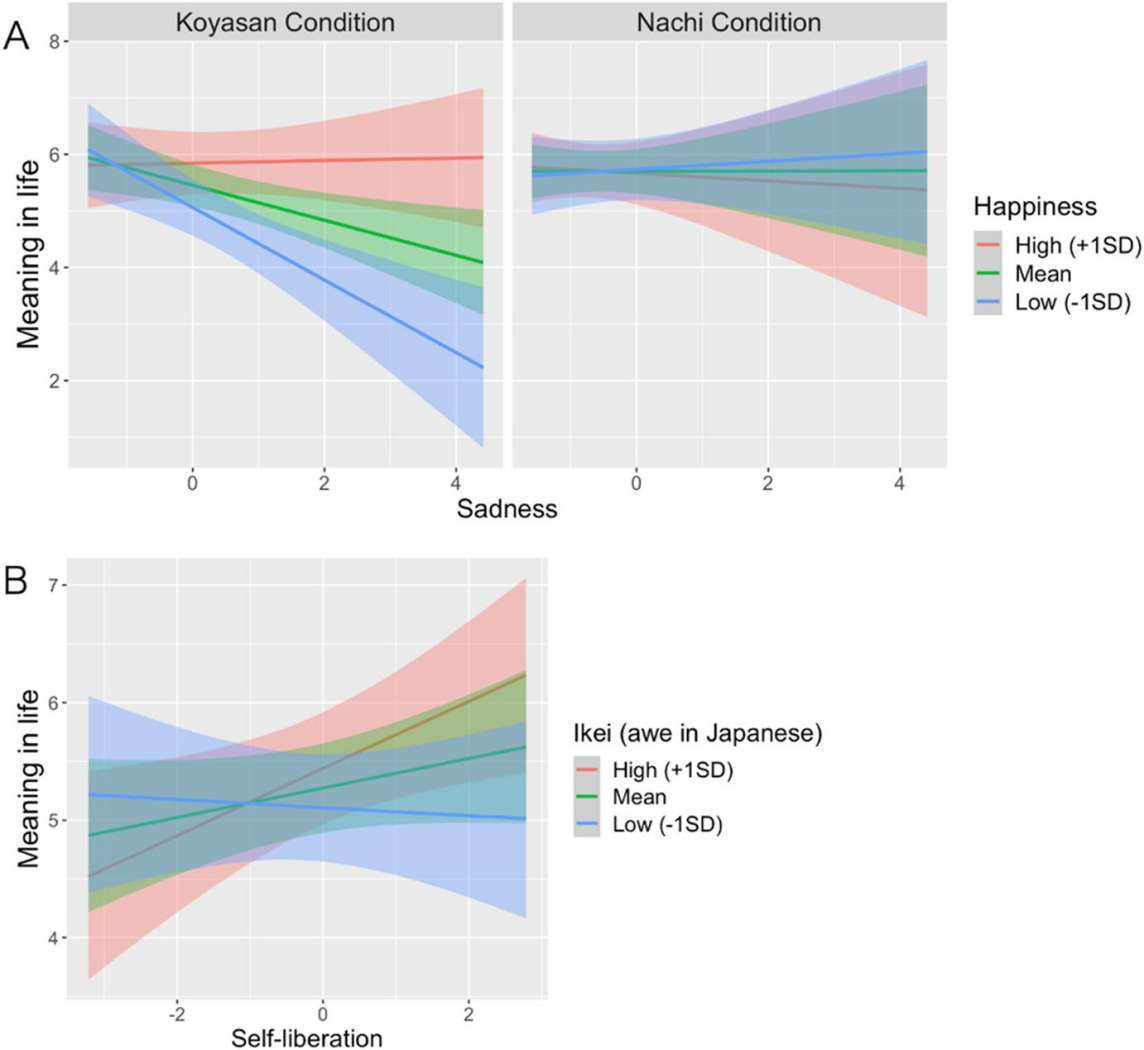
**a** Simple slope showing the role of happiness as a moderator in the relationship between the condition, sadness, and meaning in life. **b** Simple slope showing the role of Ikei (Awe in Japanese) as a moderator in the relationship between the condition and meaning in life. *Note*. Low levels were calculated at  − 1 standard deviation (SD) below the mean; high levels were calculated at  + 1 SD above the mean

Consistent with *H2*, the Bayesian linear regression model with the interactions showed that the condition × sadness × happiness interaction effect on meaning in life was significant (*b* = 0.14, 95% CI [0.03, 0.25]; Table S4). Simple slope analyses revealed that the negative relationship between sadness and meaning in life was evident among participants in the Koyasan condition who reported lower levels of happiness (− 1 SD: *b* =  − 0.64, 95% CI [− 0.98, −0.31]), but not among those who reported higher levels of happiness (+ 1 SD: *b* = 0.02, 90% CI [−0.21, 0.24]; Fig. 2a). This relationship was not significant in the Nachi condition, regardless of the ratings of happiness (− 1 SD: *b* = 0.06, 90% CI [− 0.21, 0.35], + 1 SD: *b* =  − 0.08, 90% CI [− 0.44, 0.27]).

In addition, in line with *H3*, the *ikei* × self-liberation interaction effect on meaning in life was significant (*b* = 0.09, 95% CI [0.00, 0.19]; Table S5). As shown in Fig. 2b, simple slope analyses revealed that the positive relationship between self-liberation and meaning in life was evident among participants who reported higher levels of *ikei* (+ 1 SD: *b* = 0.28, 95% CI [0.09, 0.49]), but not among those who reported lower levels of *ikei* (− 1 SD: *b* = − 0.04, 90% CI [− 0.23, 0.16]). Controlling for gender and religious affiliation, none of these results changed meaningfully except the mean difference of meaning in life between conditions (*b* =  − 0.22, 90% CI [− 0.46, 0.03]). Moreover, correlation analyses showed that the relationship between meaning in life or self-liberation and *ikei* or *ifu* was not significant in each condition (|*r*s| < 0.20, *p*s > 0.18). Similar results were obtained for samples with the two conditions combined. Therefore, higher levels of both *ikei* and self-liberation were associated with higher ratings of meaning in life.

The text-mining analyses revealed that the most used word in both conditions was sightseeing. In each condition, 18 and 27 out of 49 participants described their purpose as “sightseeing,” respectively. Moreover, as shown in Fig. 1a and b, eight participants used death-related words (e.g., sick, dead, graves) in the Koyasan condition (for example, “visiting a grave;” “because I became sick, and I wanted to get a shuin stamp because of a ‘thank you’ visit to the priest;” “memorial for the war dead;” and “commemoration of my late husband”), while five participants used words such as “refresh” and “memories” in the Nachi condition (for example, “to make memories” and “to get refreshed”).

Further, exploratory correlation analyses showed that a positive correlation between *ikei* and *ifu*, both are awe in Japanese, was observed in the Koyasan condition (*r* = 0.50, *p* < 0.001), but not in the Nachi condition (*r* = 0.05, *p* = 0.76). The difference between these two independent correlations was significant (*Z* = 2.39, *p* = 0.002). Thus, the relationship between *ikei* and *ifu* was stronger in the Koyasan condition than in the Nachi condition.

## Discussion

This study investigated the relationships between the positive and negative sides of spirituality and meaning in life. We examined how the sense of meaning in life is associated with positive and negative spiritual experiences by conducting a field study at the Kumano Nachi Taisha shrine and the Koyasan Okunoin temple, two famous religious sites in Japan. Results showed that participants in the Koyasan condition reported a marginally lower sense of meaning in life and greater feelings of sadness than those in the Nachi condition. The negative relationship between sadness and meaning in life was evident among participants in the Koyasan condition who reported lower levels of happiness, but not among those who reported higher levels of happiness. Regardless of the condition, the positive relationship between self-liberation and meaning in life was evident among participants who reported higher levels of *ikei*. In addition, the exploratory analyses including text-mining methods showed that in the Koyasan condition, the relationship between *ikei* and *ifu* was evident and death-related words were more frequently used when describing their purpose of the visit than in the Nachi condition.

The negative spiritual experience at the Koyasan Okunoin temple was associated with a marginally lower sense of meaning in life and greater feelings of sadness. Previous studies have demonstrated that higher levels of loneliness and lower levels of self-esteem are associated with less meaning in life (Hicks & King, 2007, 2008, 2009). Moreover, feelings of sadness at the Koyasan Okunoin temple, which has several graves surrounded by nature, might be associated with the awareness of the mortality of the self, one’s family, or one’s ancestors. In fact, the text-mining analysis revealed that several participants used death-related words when describing their purpose of the visit. Given that grief—a emotional response to the loss of someone or something that was important, which includes profound sadness—increases the sense of loss of meaning, goals, and interests in life (Shear & Shair, 2005), our findings might provide new insights into the meaning in life in terms of negative aspect of spirituality.

In the Koyasan condition, the negative association between sadness and meaning in life was salient among participants who reported lower levels of happiness, but not among those who reported higher levels of happiness. These results are in line with previous findings that positive emotions play a role in protecting individuals from factors that interfere with meaning in life as they improve it even when individuals do not have a source of meaning such as religiosity or self-esteem (Hicks & King, 2007, 2008, 2009). Thus, these results suggest that positive emotions could contribute to retain meaning in life even in the presence of negative emotions.

One of the main characteristics of negative spirituality is high level of uncertainty evoked by awareness of mortality and feelings of chaos (Exline et al., 2014; Jones et al., 2015; Pargament, 1997), which enhance subsequent savoring—an emotion-regulation strategy that involves deliberately upregulating positive affect—that provides a greater sense of meaning in life (Bryant & Smith, 2015; Gregory et al., 2023). Therefore, one explanation for our findings is that positive emotions could function as an adaptive response to negative spiritual experiences. Future research should examine the relationship between positive/negative spirituality and meaning in life from the perspective of the adaptive function of emotion.

The positive relationship between self-liberation and meaning in life was evident among participants who felt greater feelings of *ikei*. Awe is related to both a non-significant sense of self and an authentic/true self (Jiang & Sedikides, 2022). Meaning in life is associated with a central self-concept that transcends the daily level (King & Hicks, 2021); thus, awe could be linked to the process of accessing a more central self-schema through its daily levels of liberation.

Interestingly, the relationship between *ikei* and *ifu*, both of which are awe in Japanese, was more evident in the Koyasan condition than the Nachi condition. While *ikei* (“畏敬”) involves respect or a positive evaluation as indicated by its second character “敬,” *ifu* (“畏怖”) conveys more negative feelings since the second character “怖” means being afraid (Takano & Nomura, 2023). Previous studies showed that *ifu* is used more frequently when describing awe experiences that involve threat (Takano & Nomura, 2023). Thus, the feelings of awe during negative spiritual experiences might be associated with threat components more strongly.

### Limitations

This study has several limitations, which could provide future research directions. First, the absence of a control condition and/or group constrains the generalizability of the findings. Spiritual feelings being rated above the midpoint indicate that the two religious sites generate experiences of spirituality; however, it is of interest to compare our findings with a daily situation or a control group of Japanese who do not have the practice of visiting religious sightseeing sites. Second, the target of this study was tourists who visited either of the two sites, neither religious practitioners nor people without interest in religion. Hence, it is unclear how the commitment to a particular religion or the religious belief systems cultivated in Koyasan and Kumano influence our findings. However, since numerous Japanese people often visit temples and shrines in their daily lives, this study enabled to identify “naïve” psychological processes in positive and negative spiritual experiences by targeting tourists at these institutions. Third, the sense of meaning in life was measured using a single item to minimize the time required to complete the survey. Previous studies have shown that meaning in life can be divided into two aspects: presence and search for meaning in life (Steger et al., 2006). Thus, future research should examine how positive and negative spiritual experiences are related to these two aspects of meaning in life. Finally, our results might be attributed to cultural characteristics. The negative aspect of spirituality in Japan might differ from monotheistic cultures since Japanese religious thoughts are polytheistic (Sugimoto, 2020). It is necessary to conduct a cross-cultural study taking the cultural differences in religious backgrounds into account.

## Conclusion

This study was the first to uncover the relationships between positive and negative spiritual experiences and meaning in life at two famous religious sites in Japan. Our results revealed similarities and differences between experiences at the sites, thereby providing important insights into the connections among spirituality, religious beliefs, and health beyond Western, educated, industrialized, rich, and democratic societies.

### Supplementary Information

Below is the link to the electronic supplementary material.

Supplementary file1 (DOCX 55 KB)

## Data Availability

Data that supported the findings of this study are publicly available in CSV format on the Open Science Framework: https://osf.io/m95pn/.

## Footnotes

1 According to the survey in 2019 (Kobayashi, 2019), although 62% of participants reported that they do not have religious faith, 74% of those believed in “gods in nature,” which is associated with nature worship. Thus, most Japanese people are thought to be spiritual but not religious (Takano & Nomura, 2022a).

2 Participants were also asked to answer items from other questionnaires (e.g., the sense of connection with friends), which will serve other, related investigations.

3 The nouns are translated from Japanese into English by professional translators.
