# Supplementary material for: Relationships Between Meaning in Life and Positive and Negative Spirituality in a Field Setting in Japan
Source: J Relig Health. 2023 Aug 19;63(1):224–37. doi: 10.1007/s10943-023-01891-8 (PMC10861397; doi:10.1007/s10943-023-01891-8)
Supplement: Supplementary file 1 — Supplementary file1 (DOCX 55 KB) [file 10943_2023_1891_MOESM1_ESM.docx]

### Supplemental Materials (Relationships between meaning in life and positive and negative spirituality in a field setting in Japan)

### Statistical Analyses

### Data analyses were conducted in R (v 4.2.3) using Bayesian linear regression models. The largest amount missing for a single variable was 5.1%; thus, missing data were handled using multiple imputation with the Multivariate Imputation by Chained Equations (MICE) package in R (van Buuren & Groothuis-Oudshoorn, 2011). We used the Markov Chain Monte Carlo (MCMC) methods for parameter estimation and the $\hat{\boldsymbol{R}}$ statistic (the Gelman–Rubin convergence statistic) to diagnose the convergence (Gelman & Rubin, 1992). Using the brms and rstan R packages (Bürkner, 2017; Carpenter et al., 2017), we obtained MCMC samples from four independent chains of 2,000 iterations, discarding the initial 1,000 samples for each chain as warm-ups. The posterior samples of 4,000 post-warmup draws indicated that models converged ($\hat{\boldsymbol{R}}$ < 1.10, bulk and tail effective sample sizes > 400).

**Table S1**.

*Mean Comparisons Between the Koyasan and Nachi Conditions on Each Variable using Bayesian Linear Regression Models and T-tests.*

| Variables | Condition | *Mean* | *SD* | Bayesian linear regression models | | | |  | *t*-tests | | |
| --- | --- | --- | --- | --- | --- | --- | --- | --- | --- | --- | --- |
|  |  |  |  | *Parameter Estimate* | *SE* | 90% CI | 95% CI |  | *t* | *p* | *d* |
| Meaning in life | Koyasan | 5.24 | 1.52 | -0.24 | 0.14 | -0.47 – -0.01 | -0.51 – 0.03 |  | -1.71 | .091 | -0.35 |
|  | Nachi | 5.71 | 1.17 |  |  |  |  |  |  |  |  |
| Self-liberation | Koyasan | 3.98 | 1.38 | -0.24 | 0.16 | -0.50 – 0.02 | -0.55 – 0.07 |  | -1.54 | .128 | -0.31 |
|  | Nachi | 4.46 | 1.68 |  |  |  |  |  |  |  |  |
| Feelings of spirituality | Koyasan | 4.46 | 1.38 | 0.00 | 0.13 | -0.21 – 0.22 | -0.25 – 0.26 |  | 0.02 | .984 | 0.00 |
|  | Nachi | 4.46 | 1.13 |  |  |  |  |  |  |  |  |
| *Ikei* | Koyasan | 4.43 | 1.65 | -0.10 | 0.18 | -0.39 – 0.19 | -0.45 – 0.24 |  | -0.60 | .553 | -0.12 |
|  | Nachi | 4.64 | 1.80 |  |  |  |  |  |  |  |  |
| *Ifu* | Koyasan | 3.44 | 1.70 | 0.28 | 0.18 | -0.01 – 0.58 | -0.07 – 0.63 |  | 1.70 | .092 | 0.35 |
|  | Nachi | 2.83 | 1.77 |  |  |  |  |  |  |  |  |
| Wonder | Koyasan | 4.18 | 1.51 | -0.14 | 0.17 | -0.42 – 0.14 | -0.47 – 0.19 |  | -0.90 | .370 | -0.18 |
|  | Nachi | 4.48 | 1.71 |  |  |  |  |  |  |  |  |
| Sadness | Koyasan | 3.06 | 1.72 | 0.48 | 0.16 | 0.22 – 0.74 | 0.18 – 0.79 |  | 3.11 | .003 | 0.63 |
|  | Nachi | 2.10 | 1.28 |  |  |  |  |  |  |  |  |
| Happiness | Koyasan | 5.16 | 1.45 | -0.18 | 0.15 | -0.44 – 0.07 | -0.49 – 0.11 |  | -1.19 | .236 | -0.24 |
|  | Nachi | 5.52 | 1.50 |  |  |  |  |  |  |  |  |
| Amusement | Koyasan | 4.98 | 1.39 | -0.16 | 0.14 | -0.38 – 0.06 | -0.43 – 0.11 |  | -1.16 | .249 | -0.24 |
|  | Nachi | 5.29 | 1.25 |  |  |  |  |  |  |  |  |
| Angry | Koyasan | 2.35 | 1.73 | 0.18 | 0.16 | -0.07 – 0.44 | -0.13 – 0.49 |  | 1.12 | .264 | 0.23 |
|  | Nachi | 2.00 | 1.29 |  |  |  |  |  |  |  |  |
| Anxiety | Koyasan | 2.58 | 1.78 | 0.10 | 0.17 | -0.18 – 0.39 | -0.24 – 0.44 |  | 0.55 | .584 | 0.11 |
|  | Nachi | 2.40 | 1.55 |  |  |  |  |  |  |  |  |
| Fear | Koyasan | 2.53 | 1.66 | 0.16 | 0.17 | -0.12 – 0.43 | -0.17 – 0.48 |  | 0.98 | .330 | 0.20 |
|  | Nachi | 2.21 | 1.58 |  |  |  |  |  |  |  |  |
| *Note*. CI = Credible Interval for the *Parameter Estimate*. | | | | |  |  |  |  |  |  |  |

**Table S2.**

*Zero-order Correlations among Main Variables (Koyasan Condition)*

|  | Variable | 1 | | 2 | | 3 | | 4 | | 5 | | 6 | | 7 | | 8 | | 9 | | 10 | | 11 | |
| --- | --- | --- | --- | --- | --- | --- | --- | --- | --- | --- | --- | --- | --- | --- | --- | --- | --- | --- | --- | --- | --- | --- | --- |
| 1 | Meaning in Life |  |  |  |  |  |  |  |  |  |  |  |  |  |  |  |  |  |  |  |  |  |  |
| 2 | Self-liberation | .19 |  |  |  |  |  |  |  |  |  |  |  |  |  |  |  |  |  |  |  |  |  |
| 3 | Feelings of Spirituality | .41 | * | .42 | ** |  |  |  |  |  |  |  |  |  |  |  |  |  |  |  |  |  |  |
| 4 | *Ikei* | .13 |  | .06 |  | .23 |  |  |  |  |  |  |  |  |  |  |  |  |  |  |  |  |  |
| 5 | *Ifu* | -.07 |  | .03 |  | .14 |  | .50 | ** |  |  |  |  |  |  |  |  |  |  |  |  |  |  |
| 6 | Wonder | .23 |  | -.22 |  | -.04 |  | .20 |  | .15 |  |  |  |  |  |  |  |  |  |  |  |  |  |
| 7 | Sadness | -.28 |  | -.34 | * | -.24 |  | -.16 |  | .11 |  | .04 |  |  |  |  |  |  |  |  |  |  |  |
| 8 | Happiness | .32 | * | .37 | * | .33 | * | -.04 |  | .14 |  | .04 |  | .00 |  |  |  |  |  |  |  |  |  |
| 9 | Amusement | .36 | * | .32 | * | .46 | ** | -.07 |  | .12 |  | .12 |  | -.13 |  | .53 | ** |  |  |  |  |  |  |
| 10 | Angry | -.11 |  | -.29 | * | -.33 | * | -.19 |  | .18 |  | .10 |  | .55 | ** | .00 |  | .01 |  |  |  |  |  |
| 11 | Anxiety | -.15 |  | -.08 |  | -.01 |  | .05 |  | .41 | ** | -.15 |  | .39 | * | -.03 |  | -.06 |  | .64 | ** |  |  |
| 12 | Fear | -.09 |  | .00 |  | .00 |  | .10 |  | .43 | ** | -.21 |  | .24 |  | -.17 |  | .07 |  | .62 | ** | .85 | ** |

*Note.* * indicates *p* < .05. ** indicates *p* < .01.

**Table S3.**

*Zero-order Correlations among Main Variables (Nachi Condition)*

|  | Variable | 1 | | 2 | | 3 | | 4 | | 5 | | 6 | | 7 | | 8 | | 9 | | 10 | | 11 | |
| --- | --- | --- | --- | --- | --- | --- | --- | --- | --- | --- | --- | --- | --- | --- | --- | --- | --- | --- | --- | --- | --- | --- | --- |
| 1 | Meaning in Life |  |  |  |  |  |  |  |  |  |  |  |  |  |  |  |  |  |  |  |  |  |  |
| 2 | Self-liberation | .09 |  |  |  |  |  |  |  |  |  |  |  |  |  |  |  |  |  |  |  |  |  |
| 3 | Feelings of Spirituality | .23 |  | .60 | ** |  |  |  |  |  |  |  |  |  |  |  |  |  |  |  |  |  |  |
| 4 | *Ikei* | .14 |  | .00 |  | .22 |  |  |  |  |  |  |  |  |  |  |  |  |  |  |  |  |  |
| 5 | *Ifu* | -.20 |  | -.04 |  | -.13 |  | .05 |  |  |  |  |  |  |  |  |  |  |  |  |  |  |  |
| 6 | Wonder | .00 |  | -.20 |  | .02 |  | .54 | ** | .03 |  |  |  |  |  |  |  |  |  |  |  |  |  |
| 7 | Sadness | .02 |  | .23 |  | .03 |  | -.24 |  | .31 | * | -.13 |  |  |  |  |  |  |  |  |  |  |  |
| 8 | Happiness | .00 |  | -.15 |  | .03 |  | .43 | ** | -.01 |  | .32 | * | -.17 |  |  |  |  |  |  |  |  |  |
| 9 | Amusement | .07 |  | -.13 |  | -.05 |  | .47 | ** | .01 |  | .45 | ** | -.13 |  | .86 | ** |  |  |  |  |  |  |
| 10 | Angry | -.10 |  | .18 |  | -.11 |  | -.21 |  | .35 | * | -.09 |  | .80 | ** | -.12 |  | -.05 |  |  |  |  |  |
| 11 | Anxiety | -.03 |  | .14 |  | -.14 |  | -.25 |  | .41 | ** | -.09 |  | .75 | ** | -.11 |  | -.03 |  | .72 | ** |  |  |
| 12 | Fear | -.06 |  | .20 |  | -.04 |  | -.25 |  | .53 | ** | -.09 |  | .76 | ** | -.06 |  | -.03 |  | .68 | ** | .82 | ** |

*Note.* * indicates *p* < .05. ** indicates *p* < .01.

**Table S4.**

*Bayesian Linear Regression Models of Condition, Sadness, Happiness, and Their Interactions on Meaning in Life*

|  | *Parameter Estimate* | *SE* | 90% CI | 95% CI |
| --- | --- | --- | --- | --- |
| Condition | -0.12 | 0.14 | -0.35 – 0.11 | -0.39 – 0.16 |
| Sadness | -0.16 | 0.09 | -0.31 – -0.00 | -0.34 – 0.03 |
| Happiness | 0.12 | 0.09 | -0.03 – 0.27 | -0.06 – 0.30 |
| Condition*Sadness | -0.15 | 0.09 | -0.30 – 0.00 | -0.33 – 0.03 |
| Condition*Happiness | 0.15 | 0.09 | -0.00 – 0.29 | -0.03 – 0.32 |
| Sadness*Happiness | 0.09 | 0.06 | -0.00 – 0.18 | -0.02 – 0.20 |
| Condition*Sadness*Happiness | 0.14 | 0.06 | 0.04 – 0.23 | 0.03 – 0.25 |
| *Note*. CI = Credible Interval for the *Parameter Estimate*. | | |  |  |

**Table S5.**

*Bayesian Linear Regression Models of Condition, Ikei (Awe in Japanese), Self-liberation, and Their Interactions on Meaning in Life*

|  | *Parameter Estimate* | *SE* | 90% CI | 95% CI |
| --- | --- | --- | --- | --- |
| Condition | -0.20 | 0.14 | -0.43 – 0.02 | -0.48 – 0.07 |
| *Ikei* (awe in Japanese) | 0.10 | 0.08 | -0.04 – 0.23 | -0.06 – 0.26 |
| Self-liberation | 0.12 | 0.09 | -0.03 – 0.27 | -0.06 – 0.30 |
| *Ikei**Self-liberation | 0.09 | 0.05 | 0.02 – 0.17 | 0.00 – 0.19 |
| *Note*. CI = Credible Interval for the *Parameter Estimate*. | | | |  |

**Table S6**.

*Predicting Meaning in Life from Condition, Sadness, Happiness, and Their Interactions*

|  | Meaning in life | | | |
| --- | --- | --- | --- | --- |
|  | β | 95% CI | *t* | *p*-value |
| Condition | -0.18 | -0.58 – 0.21 | -0.92 | .361 |
| Sadness | -0.18 | -0.39 – 0.03 | -1.68 | .096 |
| Happiness | 0.13 | -0.06 – 0.33 | 1.33 | .185 |
| Condition x Sadness | -0.35 | -0.78 – 0.08 | -1.62 | .109 |
| Condition x Happiness | 0.31 | -0.08 – 0.70 | 1.58 | .118 |
| Sadness x Happiness | 0.15 | -0.03 – 0.34 | 1.61 | .110 |
| Condition x Sadness x Happiness | 0.47 | 0.09 – 0.84 | 2.48 | .015 |
| *Note*. CI = Confidence Interval for the standardized β. | | |  |  |

**Table S7**.

*Predicting Meaning in Life from Condition, Self-liberation, Ikei, and Their Interaction*

|  | Meaning in life | | | |
| --- | --- | --- | --- | --- |
|  | β | 95% CI | *t* | *p*-value |
| Self-liberation | 0.12 | -0.08 – 0.32 | 1.16 | .248 |
| *Ikei* | 0.14 | -0.06 – 0.34 | 1.37 | .174 |
| Self-liberation x *Ikei* | 0.19 | 0.02 – 0.37 | 2.16 | .034 |
| Condition | -0.29 | -0.69 – 0.11 | -1.42 | .159 |
| *Note*. CI = Confidence Interval for the standardized β. | | |  |  |

**References**

Bürkner, P.-C. (2017). brms: An R Package for Bayesian Multilevel Models Using Stan. *Journal of Statistical Software*, *80*, 1–28. https://doi.org/10.18637/jss.v080.i01

Carpenter, B., Gelman, A., Hoffman, M. D., Lee, D., Goodrich, B., Betancourt, M., Brubaker, M. A., Guo, J., Li, P., & Riddell, A. (2017). Stan: A probabilistic programming language. *Journal of Statistical Software*, *76*, 1–32. https://doi.org/10.18637/jss.v076.i01

Gelman, A., & Rubin, D. B. (1992). Inference from Iterative Simulation Using Multiple Sequences. *Statistical Science: A Review Journal of the Institute of Mathematical Statistics*, *7*(4), 457–472. http://www.jstor.org/stable/2246093

van Buuren, S., & Groothuis-Oudshoorn, K. (2011). mice: Multivariate Imputation by Chained Equations in R. *Journal of Statistical Software*, *45*, 1–67. https://doi.org/10.18637/jss.v045.i03
